# Delivering Benefits at Speed Through Real-World Repurposing of Off-Patent Drugs: The COVID-19 Pandemic as a Case in Point

**DOI:** 10.2196/19199

**Published:** 2020-05-13

**Authors:** Moshe Rogosnitzky, Esther Berkowitz, Alejandro R Jadad

**Affiliations:** 1 MedInsight Research Institute Rehovot Israel; 2 Program in Impactful Giving Dalla Lana School of Public Health University of Toronto Toronto, ON Canada; 3 Department of Anesthesiology and Pain Medicine Faculty of Medicine University of Toronto Toronto, ON Canada

**Keywords:** COVID-19, drug costs, drug repositioning, drugs, generic, off-label use, public health, severe acute respiratory syndrome coronavirus 2, pandemic, crisis

## Abstract

Real-world drug repurposing—the immediate “off-label” prescribing of drugs to address urgent clinical needs—is a widely overlooked opportunity. Off-label prescribing (ie, for a nonapproved indication) is legal in most countries and tends to shift the burden of liability and cost to physicians and patients, respectively. Nevertheless, health crises may mean that real-world repurposing is the only realistic source for solutions. Optimal real-world repurposing requires a track record of safety, affordability, and access for drug candidates. Although thousands of such drugs are already available, there is no central repository of off-label uses to facilitate immediate identification and selection of potentially useful interventions during public health crises. Using the current coronavirus disease (COVID-19) pandemic as an example, we provide a glimpse of the extensive literature that supports the rationale behind six generic drugs, in four classes, all of which are affordable, supported by decades of safety data, and targeted toward the underlying pathophysiology that makes COVID-19 so deadly. This paper briefly summarizes why cimetidine or famotidine, dipyridamole, fenofibrate or bezafibrate, and sildenafil citrate are worth considering for patients with COVID-19. Clinical trials to assess efficacy are already underway for famotidine, dipyridamole, and sildenafil, and further trials of all these agents will be important in due course. These examples also reveal the unlimited opportunity to future-proof our health care systems by proactively mining, synthesizing, cataloging, and evaluating the off-label treatment opportunities of thousands of safe, well-established, and affordable generic drugs.

December 2019 heralded the transformation of modern-day life. A new and lethal disease, now named COVID-19, was emerging in China and was about to change the world as we know it. The same month, in propitious timing, a few hundred of the world’s leading physicians, scientists, government agency officials, and nonprofit leaders gathered at an inaugural 2-day conference jointly sponsored by the US Food and Drug Administration (FDA) and National Institutes of Health (NIH) in Washington, DC. The topic of the conference was “Repurposing Off-Patent Drugs,” and attendees had convened to discuss how widely used, low-cost, and safe medicines that are approved for one indication might be harnessed to provide additional, novel, and sometimes unexpected therapeutic benefits in other diseases.

Dr Christopher Austin, Director of the National Center for Advancing Translational Sciences at the NIH, opened the conference by welcoming the birth of a new era in human medicine. He asked participants “to skewer some sacred cows,” emphasizing the need to embrace controversial thinking to improve patients’ lives.

Drug repurposing seems tantalizingly simple. Conservatively, there are 6,500 human diseases that have no regulatory-approved treatments whatsoever. At the current rate of progress, it will be 2,000 years before every human disease is treatable. What percentage of those 6,500 currently untreatable diseases is ameliorable, to some degree, by a drug you can get at [your local pharmacy]? Shame on us if we can’t figure out a way to make these available to patients suffering from disabling and lethal diseases. This is an eminently solvable problem.

If drug repurposing was an obscure subject for experts as well as the public, COVID-19 has changed that forever. The publicity generated by the US president endorsing the antimalarial agents hydroxychloroquine and chloroquine as treatments for COVID-19 jolted regulatory authorities worldwide. The FDA felt compelled to grant emergency-use authorization for these drugs, while the European Medicines Agency held back, urging that they should not be prescribed outside of clinical trials and nationally agreed upon protocols. In the absence of proven treatments, many physicians at the frontlines of the COVID-19 battle prescribed these drugs, resulting in a worldwide shortage. Conflicting clinical trial data have emerged since then regarding use of these antimalarial drugs in COVID-19 [1-7], some of which indicate a lack of benefit or even the potential for harm [6]. This underscores the need for emergency regulatory authorization of unproven treatments, if deemed necessary in a public health crisis, to be based first and foremost on robust evidence of safety. It is also important that the relevant agency issues a statement emphasizing the exploratory nature of the intervention and urgent need for robust clinical trial data to support ongoing use.

Hydroxychloroquine and chloroquine were developed as antimalarial treatments and subsequently repurposed for treating systemic lupus erythematosus and rheumatoid arthritis. Their repurposing for these challenging autoimmune diseases was facilitated by funding from pharmaceutical companies, which recouped their investment through patent-protected revenues until the drugs became available as generics. However, only a small proportion of drug-repurposing discoveries enjoy patent protection and can benefit from the large and costly clinical trials necessary for regulatory approval.

By contrast, real-world repurposing—the immediate “off-label” prescribing of drugs by caring physicians based on their acumen, awareness of pilot studies or case reports, or field experience in the clinical setting—is a widely overlooked opportunity. Prescribing a drug off-label (ie, for a use other than what it was approved for) is legal in almost every country worldwide. However, if there is an unforeseen adverse outcome, the burden of liability shifts from the regulator or pharmaceutical company to the prescribing physician. Additionally, the burden of payment shifts from the insurer or other institutional health care payers to the patient. Nevertheless, when dealing with immediate and urgent health crises, whether at an individual or public level, real-world repurposing is frequently the only realistic solution.

To protect the public from unscrupulous players, the US FDA prohibits pharmaceutical companies from promoting off-label uses of their drugs, which could be used to increase profit while avoiding investment in clinical trials. By contrast, the FDA is supportive of disseminating information about promising off-label uses by independent entities, a point reiterated in March 2020 on the FDA’s website [8]. This underscores the importance of vigorous efforts to create reliable, independent evidentiary repositories to disseminate such treatment opportunities, and thereby support the decision making of those in the frontlines, in nearly real time.

Two additional critical elements are prerequisites if real-world repurposing is to deliver health benefits at the public level: safety and affordability. The former calls for a decades-long track record of established safety, and the latter requires the availability of generic low-cost drug candidates. Fortunately, many thousands of such drugs are already available. The challenge is that no central repository of off-label uses exists in a way that enables immediate intervention in times of public health crises.

Taking the COVID-19 pandemic as an example, we have selected four well-established drugs backed by many decades of safety data, widespread use, and affordability, which we believe offer the opportunity to prevent or treat both the viral infection and the disabling and deadly complications that ensue. Although COVID-19 usually presents with respiratory symptoms, infection that spreads beyond the lung contributes significantly to the disease toll through uncontrolled outpouring of immune cells, disturbed clotting, multi-organ failure, and other life-threatening complications. There is extensive clinical support, backed by a solid mechanistic scientific rationale, underpinning the proposed drugs (Multimedia Appendix 1). Each was selected based on safety, affordability, and ability to target multiple aspects of the underlying disease processes that make COVID-19 so deadly. The proposed doses are those that have been shown to achieve the target physiological effects as demonstrated in the supporting references.

Cimetidine and famotidine, which are approved for heartburn caused by reflux disease [9], have been shown to have powerful effects on the immune system [10]. Data indicate that they can suppress a wide variety of common viruses, including herpes and human papillomaviruses [11-13], and boost immune response after vaccination [14-20], with additional immune-modulating effects in a range of cancers and allergic diseases [10]. They have also shown efficacy in protecting the heart from excessive workload, lowering blood pressure, and improving cardiac efficiency [21,22]; reducing inflammation [23]; and inhibiting pathological blood clotting [24,25]. A clinical trial of famotidine in COVID-19 was started recently in New York, following the observation (as yet unpublished) that certain patients in China who were taking it when diagnosed with COVID-19 had better clinical outcomes than those who were not [26]. Data generated from this new study are eagerly awaited.

The antiplatelet agent dipyridamole, which is approved to prevent thrombotic events in at-risk patients [27,28], has also caught the eye of researchers investigating potential treatments for COVID-19. A recently published study in China illustrated its ability to suppress the severe acute respiratory syndrome coronavirus 2 virus that causes COVID-19, leading to marked clinical improvements [29]. A larger study recently launched in China examines dipyridamole in 460 patients with COVID-19 (ChiCTR2000030055). Beyond these antiviral effects, dipyridamole has shown anti-inflammatory, antioxidant, and vasodilatory activity [30-34], and is one component of a widely used anticoagulant (citrate-theophylline-adenosine-dipyridamole [CTAD]) [35-37]. Clinically, cardioprotective effects have been reported in patients with chronic heart failure [38], and improved renal function is documented in patients with chronic kidney disease, delaying risk of progression to dialysis and reducing mortality [39,40].

The cholesterol-lowering agents fenofibrate and bezafibrate are approved for treatment of dyslipidemias [41]. Although bezafibrate is unavailable in the United States, it is widely used in Europe. Meta-analyses show that they can reduce disability and death from atherosclerotic cardiovascular disease and stroke, independent from their effects on cholesterol [42,43]. Potentially protective effects on kidney function have been reported [44,45], along with antiviral efficacy in patients with a hepatitis C virus infection [46]. In some patients, fibrates have lowered plasma fibrinogen levels to a statistically significant degree [47-52], suggesting the potential to address the dangerous hypercoagulability seen in many patients with COVID-19. Indeed, fibrates have demonstrated anticoagulant and cardiovascular protective effects in patients with metabolic syndrome [53], which represents a hypercoagulable state accompanied by inflammation and endothelial dysfunction.

The phosphodiesterase-5 (PDE-5) inhibitor sildenafil citrate is a vasodilator that was approved in 1998 for treating erectile dysfunction [54] and more recently received an indication for pulmonary arterial hypertension (PAH) [55]. Sildenafil has a wide range of anti-inflammatory, antioxidant, and vasodilatory actions across many body systems, with benefits reported in case studies of patients with type 2 diabetes [56,57] and hematological cancers [58]. Reported cardioprotective effects, stemming from improved pulmonary circulation as well as direct action on the myocardium [59], include improved cardiac contractility and reduced symptoms in patients with a range of cardiac disorders [60-62], with reduction in cardiovascular events and mortality in patients at high risk [63]. Studies demonstrating sildenafil’s efficacy and tolerability in PAH continue to accrue, and a recent Cochrane review and meta-analysis concluded that patients with PAH who received PDE-5 inhibitors were significantly less likely to die in the short-term than those receiving a placebo [64]. Sildenafil may also reduce mortality in idiopathic pulmonary fibrosis [65], an interstitial lung disease with high mortality, and preliminary evidence suggests that this drug class is actively renoprotective [62,66]. Sildenafil is currently under investigation in a phase 3 trial in patients with COVID-19 (NCT04304313), which will help clarify its therapeutic potential.

Times of emergency, such as with the COVID-19 pandemic, call for a radical review of the way we practice medicine. As Dr Austin aptly stated, we have to be ready “to skewer some sacred cows.” Clinical trials of unprofitable generic drugs sponsored by governments or nonprofit organizations are obviously welcome and important but should not delay the judicious use of well-established, safe, cost-effective, and rationally prescribed therapies.

The race to find a cure for COVID-19 has resulted in unprecedented worldwide research efforts. As of the time of writing, the Milken Foundation has compiled a list of treatments being studied for COVID-19 [67]. Nevertheless, the time to approval and the expected high cost of the majority of these drugs may leave them out of reach for a large portion of the world’s population.

The four well-established drugs presented here for consideration, alone or in combination, for at-risk patients with COVID-19 highlight the gems buried in the mountain of hundreds of thousands of clinical studies, inaccessible to physicians battling at the frontlines of clinical medicine. Unbeknownst to most of them, the four drugs selected in this case, officially approved for a handful of indications, have shown efficacy in managing over 100 additional diseases. We do not propose specifically when or how each of these drugs should be used; rather, we aim to provide a pathophysiological rationale for their use, alone or in combination; share our understanding of why and how they may provide benefit; and spur creative thinking about their potential use in this disease while illustrating the untapped potential of therapeutic options that may be hidden in plain sight.

The COVID-19 pandemic represents an unparalleled opportunity to refocus our efforts on mining, synthesizing, and cataloging the body of evidence behind many promising treatment opportunities. This article is an invitation to kindred spirits and curious, bold humanitarians to pool efforts to harness this opportunity to future-proof our health care systems based on robust science. We owe it to ourselves and future generations.

## Appendix

Multimedia Appendix 1 Approved indications and recognized physiological effects of drugs to consider repurposing for patients with COVID-19.

## Abbreviations

COVID-19: coronavirus disease
FDA: Food and Drug Administration
NIH: National Institutes of Health
PAH: pulmonary arterial hypertension
PDE-5: phosphodiesterase-5
