# Supplementary material for: Delivering Benefits at Speed Through Real-World Repurposing of Off-Patent Drugs: The COVID-19 Pandemic as a Case in Point
Source: JMIR Public Health Surveill. 2020 May 13;6(2):e19199. doi: 10.2196/19199 (PMC7224168; doi:10.2196/19199)
Supplement: Multimedia Appendix 1 [file publichealth_v6i2e19199_app1.docx]

| **Drug** | **Current indications** | **Proposed dose** | **Clinical research evidence of protective effects** | | | | | | **Notes** |
| --- | --- | --- | --- | --- | --- | --- | --- | --- | --- |
|  |  |  | **Anti-inflammatory** | **Anti-viral and/or anti-COVID-19** | **Anti-coagulant** | **Cardio-**  **protective** | **Reno-**  **protective** | **Lung**  **protective** |  |
| **Cimetidine or famotidine** | Symptomatic management of GERD | Cimetidine 200mg QID  OR  Famotidine 20 - 40 mg BID |  | X |  | X |  |  | - Establish baseline prolactin levels and monitor periodically. - May increase serum concentrations of other drugs. - Reduces absorption of dipyridamole |
| **Dipyridamole** | Antithrombotic | 75mg TID  OR  50-100mg once weekly | X | X^a^ | X | X | X |  | - May cause headaches during first week of use. - Taking with   foods or antacids halves absorption |
| **Fenofibrate or bezafibrate** | Dyslipidemia | Fenofibrate ≤200mg/day  OR  Bezafibrate 400mg/day | X | X | X | X | X |  | - Significant reduction in D-dimer and fibrinogen usually seen in days. |
| **Sildenafil citrate** | Erectile dysfunction | 25mg BID, on an empty stomach | X |  |  | X | X | X | - Avoid grapefruit juice (increases sildenafil levels). - Cimetidine/ famotidine may increase sildenafil concentration. If combined, consider lower sildenafil dose - even 12.5mg BID |

^a^ Preliminary evidence of efficacy against COVID-19 exists

BID, twice daily; GERD, gastroesophageal reflux disease; QID, four times daily; TID, three times daily
